# Natural vs. programmed cycles for frozen embryo transfer: study protocol for an investigator-initiated, randomized, controlled, multicenter clinical trial

**DOI:** 10.1186/s13063-021-05637-3

**Published:** 2021-09-27

**Authors:** Sheriza Baksh, Anne Casper, Mindy S. Christianson, Kate Devine, Kevin J. Doody, Stephan Ehrhardt, Karl R. Hansen, Ruth B. Lathi, Fatmata Timbo, Rebecca Usadi, Wendy Vitek, David M. Shade, James Segars, Valerie L. Baker, NatPro Study Group, Lusine Aghajanova, Ruben Alvero, Mary Andrews, Diego Arevalo, Emily Barnard, Aracely Casillas, Morgan Copeland, La Tasha B. Craig, Kathleen M. Doody, Ashley Eskew, Alma Gonzalez, Gretchen Hoelscher, Arthur Jason Vaught, Lynda Kochman, Andie Lears, Gaya Murugappan, Anna C. Nackley, Tasha Newsome, Natalie Quintana, Bhuchitra Singh, Anna Sokalska, Michelle Starkey-Scruggs, Robin L. Thomas, Deveine Toney, Irene Trueheart, Kisha Turner, Rebecca Usadi, Sally Villalobos, Anjali Wignarajah, Virginia Winn, Christy Zones

**Affiliations:** 1 Department of Epidemiology, Johns Hopkins Bloomberg School of Public Health, 415 N. Washington Street, 2nd Floor, Baltimore, MD 21231 USA; 2 Center for Clinical Trials and Evidence Synthesis, Johns Hopkins University, Baltimore, MD 21205 USA; 3 Division of Reproductive Endocrinology and Infertility, Department of Gynecology and Obstetrics, Johns Hopkins University School of Medicine, Baltimore, MD 21205 USA; 4 Shady Grove Fertility, Washington, DC 20006 USA; 5 CARE Fertility, Bedford, TX 76022 USA; 6 Section of Reproductive Endocrinoloogy and Infertility, Department of Obstetrics and Gynecology, University of Oklahoma College of Medicine, Oklahoma City, OK 73104 USA; 7 Department of Obstetrics and Gynecology, Stanford University Medical Center, Sunnyvale, CA 94087 USA; 8 Atrium Health, Charlotte, NC 28203 USA; 9 University of Rochester Medical Center, Rochester, NY 14642 USA

**Keywords:** Frozen-thawed embryo transfer, Modified natural cycle, Programmed cycle, Preeclampsia

## Abstract

**Background:**

Randomized trials of assisted reproductive technology (ART) have been designed for outcomes of clinical pregnancy or live birth and have not been powered for obstetric outcomes such as preeclampsia, critical for maternal and fetal health. ART increasingly involves frozen embryo transfer (FET). Although there are advantages of FET, multiple studies have shown that risk of preeclampsia is increased with FET compared with fresh embryo transfer, and the reason for this difference is not clear. NatPro will compare the proportion of preeclampsia between two commonly used protocols for FET,modified natural and programmed cycle.

**Methods:**

In this two-arm, parallel-group, multi-center randomized trial, NatPro will randomize 788 women to either modified natural or programmed FET and follow them for up to three FET cycles. Primary outcome will be the proportion of preeclampsia in women with a viable pregnancy assigned to a modified natural cycle FET (corpus luteum present) protocol compared to the proportion of preeclampsia in pregnant women assigned to a programmed FET (corpus luteum absent) protocol. Secondary outcomes will compare the proportion of live births and the proportion of preeclampsia with severe features between the protocols.

**Conclusion:**

This study has a potential significant impact on millions of women who pursue ART to build their families. NatPro is designed to provide clinically relevant guidance to inform patients and clinicians regarding maternal risk with programmed and modified natural cycle FET protocols. This study will also provide accurate point estimates regarding the likelihood of live birth with programmed and modified natural cycle FET.

**Trial registration:**

ClinicalTrials.govNCT04551807. Registered on September 16, 2020

**Supplementary Information:**

The online version contains supplementary material available at 10.1186/s13063-021-05637-3.

## Background

Nearly one third of the estimated 1.6 million cycles of assisted reproductive technology (ART) performed globally each year are categorized as frozen embryo transfers (FET) [1]. Given this, it is of serious concern that multiple studies show an association between FET and increased risk for hypertensive disorders of pregnancy, including preeclampsia [2–11]. Preeclampsia is characterized by elevated blood pressure and proteinuria [12] and is associated with severe short- and long-term consequences for both the mother and infant. Women experiencing preeclampsia during pregnancy can have a host of additional pregnancy complications, such as seizures, myocardial infarction, and in severe cases, hemodialysis, elevated liver enzymes, and low platelets (HELLP) syndrome [13–19]. Since the only cure for preeclampsia is delivery of the placenta, understanding possible associations between FET protocols and preeclampsia is critical to mitigating adverse maternal and fetal outcomes.

Recent findings implicate one commonly performed FET protocol as a risk factor for preeclampsia [20–24]. In a prospective cohort study, the programmed FET protocol was associated with significantly higher proportion of preeclampsia compared to the natural cycle FET protocol. Furthermore, through a detailed examination of maternal cardiovascular adaptation to pregnancy in two separate populations, perturbations of concern were seen with programmed FET, but not with natural cycle FET. This association is thought to be driven by the absence of a corpus luteum in the programmed cycle. There are some advantages to programmed cycles, including greater convenience for both the patient and the clinic.

To date, most randomized trials of ART procedures have focused on the outcomes of clinical pregnancy or live birth and have not been powered for obstetric outcomes such as preeclampsia which are critical for maternal and fetal health. We will conduct a multi-site, clinical trial to randomize women to either a modified natural or programmed FET for up to three cycles or until a pregnancy that reaches at least 20 weeks gestational age. Our primary objective will be to compare the proportion of preeclampsia in the modified natural versus programmed cycle. We hypothesize that pregnancy resulting from a modified natural cycle FET will have a lower proportion of preeclampsia compared to that of a programmed FET and that the proportion of live births will be similar between arms. All embryos will be created prior to enrollment in the trial, and no embryo will be subject to increased risk in the course of this research.

## Methods

### Overview of study design

NatPro is a two-arm, parallel-group, multi-center, randomized trial, investigating the association between modified natural and programmed FET with preeclampsia. We will recruit 788 women from seven nationwide clinical centers for randomization to either the modified natural or programmed FET arm. Each study participant will have up to three FET cycles to experience a live birth in the study. The primary outcome in NatPro is the proportion of preeclampsia or maternal or fetal death in pregnant women assigned to a modified natural cycle FET protocol compared to the proportion in pregnant women assigned to a programmed FET protocol. The presence of preeclampsia will be assessed by a masked adjudication board, using the most recent criteria for hypertensive disorders in pregnancy from The American College of Obstetrics and Gynecology.

### Study oversight, recruitment, and eligibility

NatPro is funded by the National Institutes of Health/National Institute of Child Health and Human Development. The NatPro protocol has been reviewed and approved by the single institutional review board at The Johns Hopkins University School of Medicine. Study monitoring will be conducted by an investigator-appointed data and safety monitoring board (DSMB). The DSMB will monitor the conduct of the trial and make appropriate recommendations to the NatPro Steering Committee regarding the continuation of the trial, safety concerns for study participants, and trial conduct.

Spanish- and English-speaking study participants will be recruited from fertility clinics at study sites across the USA. Potential participants will be recruited via medical records review, communication with clinic staff, and written materials, such as flyers and pamphlets, posted in the study clinics. All personal identifiers will be housed locally in patient medical records at study clinics and will not be data entered for NatPro.

The following inclusion criteria are required of all NatPro participants. All participants must plan to undergo FET with an embryo that was created with autologous oocytes with a planned transfer to the participant’s uterus. The embryos must have been created when the participant was age 18-39 if the embryo did not undergo PGT testing for aneuploidy, and study enrollment must occur while the participant’s age is less than 42 years. If PGT testing indicates that euploid embryos were created, the participant may transfer embryos created when the participant was between the ages of 18–41. Each participant with PGT testing results must have at least one vitrified blastocyst with euploid result or, if no PGT testing results available, a vitrified blastocyst of fair or better quality available for transfer. In addition to these screening criteria, participants must meet the following criteria: body mass index ≤ 40, a normal uterine cavity screen within 1 year of enrollment, a menstrual cycle length of 24–35 days, normal TSH, and for women with a BMI > 30, a normal hemoglobin A1c within the past year. A full listing of eligibility criteria are listed in Supplemental Table 1.

### Study flow, randomization, and masking

**Fig. 1:**
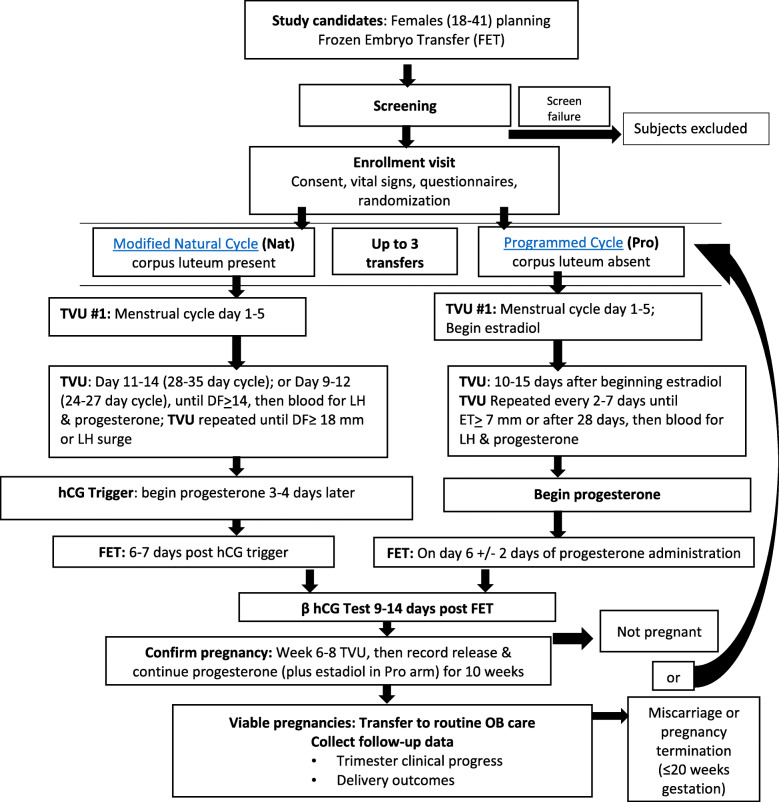
Overview of patient flow, study procedures, and outcomes

Study procedures are shown in Fig. 1. NatPro participants will be randomized through the use of a concealed, remote, computer-generated algorithm to receive either the modified natural or programmed cycle FET. They will be allowed to complete up to three cycles in their randomized treatment arm until pregnancy up to 20 weeks gestation within a 2-year period. If a participant has a biochemical pregnancy, miscarriage, ectopic pregnancy, or pregnancy termination before 20 weeks gestation, she will be permitted to continue in the protocol and begin another FET cycle, not to exceed three cycles. Women experiencing pregnancy loss > 20 weeks gestation will not begin another FET cycle in the study. Once women are released to obstetrical care, investigators will follow them via trimester and post-pregnancy telephone contacts and medical record collection.

Between randomization and FET, women will be asked to complete a second set of questionnaires, “Pittsburgh Sleep Quality Index” [25], “FertiQoL” [26], and an instrument to assess participant satisfaction with study procedures. During the trimester and post-pregnancy calls, sites will collect data on pregnancy status, medications, treatments, and adverse events. Blood draws and urine specimens will be collected from all women at baseline and 6–8 weeks gestation, and optionally at later time points, for analysis of hormones, proteomics, lipidomics, metabolomics, and other potential markers of preeclampsia or other pregnancy complications. Data collection is further detailed in Table 1.

**Table 1:** Data collection and procedures by visit

	Screening/enrollment	FET cycle		Post FET	Pregnancy confirmation OB scan	Pregnancy follow-up
				9–14 days post FET	6–8 weeks	Each trimester and delivery
		Modified natural	Programmed			
Informed consent	X					
Eligibility determination	X					
Demographics	X					
Medical, reproductive, and obstetric history	X					
Medication reconciliation	X	X	X	X	X	X
Vital signs	X				X	X
Physical exam	X					
PAP smear will be recommended to be done with gynecologist if not done according to ACOG guidelines	X					
Ultrasound (transvaginal while at study site)		X	X		X	X
Estradiol medication self-administration (programmed cycle only)			X	X	X	
hCG self-administration (medication to trigger ovulation)		X				
Progesterone medication self-administration		X	X	X	X	
Embryo transfer		X	X			
Questionnaires	X			X		
Randomization	X					
Adverse event monitoring		X	X	X	X	X
Medical record release					X	
Pregnancy data collection						X
Delivery data collection						X
Neonatal data collection						X
Blood and urine samples for banking at study site (baseline scan of first cycle, 6–8 weeks pregnancy visit)		X	X		X	
Blood draw(s) for hormone levels used in cycle monitoring		X	X			
Optional specimen collection at study site						X

The data coordinating center (DCC) will generate treatment assignment schedules using permuted blocks of varying length, stratified by clinical center and PGT status. While all participants, clinical staff, site investigators, and the principal investigators will be unaware of the allocation sequence, they will be unmasked to treatment assignment. Only DCC staff involved in the allocation of treatment assignments will be aware of the allocation sequence. The primary outcome adjudication board will be masked to treatment assignments. All study data will be stored on secure, remote servers, accessible by certified personnel only.

### Interventions

Study procedures are within standard of care for both treatment arms across sites, with some specifics such as the choice of estradiol and progesterone used in the programmed arm, at the discretion of the treating physician (Fig. 1). To confirm the presence or absence of the corpus luteum, we will employ detailed sonographic and hormone monitoring throughout both cycle protocols. Participants will be randomized to either the modified natural cycle, which includes ovulation and creation of the corpus luteum or to the programmed cycle, which suppresses ovulation with no formation of the corpus luteum. After confirmation of a viable pregnancy at approximately 6–8 weeks gestational age, study participants in both treatment arms will be released to obstetrical care for routine pregnancy follow-up.

The criteria for cycle cancelation include: (1) failure to develop a follicle of ≥ 14 mm by day 28 (modified natural), (2) collapse of the lead follicle if the specific initial day of follicle collapse cannot be known (modified natural), (3) persistent fluid in the endometrial cavity, (4) development of abnormality in the endometrium not previously noted, (5) failure to achieve endometrial thickness of ≥ 5 mm, or (6) serum progesterone level approximately ≥ 1.5 ng/ml on the day of the last monitoring ultrasound (programmed). If embryo transfer does not occur, the canceled cycle will not be counted as one of the three FET cycles in the study for the participant.

#### Modified natural cycle protocol

This treatment protocol is defined as a modified natural cycle where the participant will administer hCG to assist with timing of the embryo transfer and supplement the luteal phase with progesterone [27]. Administration of hCG will trigger ovulation and prevent complete reliance on detection of the spontaneous luteinizing hormone (LH) surge.

Modified natural cycle FET requires ultrasound monitoring and blood hormonal measurements for optimal timing of the hCG trigger [27]. Commonly, participants will typically have up to 3 ultrasounds prior to hCG administration and embryo transfer. The first of these is a transvaginal ultrasound at baseline on days 1–5. This ultrasound will serve as a comparator for later follicle development. At this visit, a blood sample will be collected for hormonal measurements, lipid profiles, and apolipoprotein levels. Study participants will also have a urine sample collection for metabolomic analysis.

The second ultrasound will be performed on cycle days 11–14 for participants with usual cycle length of 28–35 days. If a participant has a cycle length of 24–25 days, the second ultrasound will be performed on cycle days 9–10. When a dominant follicle reaches at least 14–16 mm, serum LH, and serum progesterone will be drawn. If there is no LH surge based on local laboratory standards (i.e. < 20 IU) or progesterone is approximately < 1.5 ng/ml, a follow-up ultrasound will be scheduled in 1–2 days. If needed, additional ultrasounds will be performed until the dominant follicle reaches at least 18–20 mm.

The hCG administration and subsequent embryo transfer will be based on current recommendations [28]. There are two scenarios under which the hCG can be administered. First, if the participant has a dominant follicle of at least 18–20 mm and serum LH < ~ 20, she will be instructed to administer hCG in the evening and the embryo transfer will be scheduled 7 days later, within a window of ± 2 days if recommended by the study physician. Second, if the participant has a serum LH surge with dominant follicle size of at least 14 mm, she will be instructed to administer hCG as soon as practical after the test result becomes available and the embryo transfer will be scheduled 6 days after the LH level exceeds 20 IU/L, within a window of ± 2 days if recommended by the study physician.

The participant will begin progesterone (most commonly vaginal progesterone 100–200 mg twice daily) 4 days after the administration of hCG if LH < 20 and 3 days after hCG administration if there is no LH surge. She will continue with progesterone treatment until the pregnancy test. If the participant is not pregnant, she will discontinue progesterone; however, if she is pregnant, progesterone will be continued without dose change until approximately 10 weeks gestation ± 2 weeks at the discretion of the study physician. Progesterone formulation will be chosen based on patient preference and physician discretion.

#### Programmed cycle protocol

In the programmed cycle, the endometrial development occurs only in association with administration of estradiol and progesterone. No ovulation occurs due to the suppression of follicle development by the estradiol administration, and the timing of the transfer is based on the number of days elapsed following initiation of exogenous progesterone. Intramuscular progesterone will be the route most commonly used in this arm as recent data support superiority with regard to ongoing pregnancy of this route of administration over vaginal progesterone in the absence of the corpus luteum [29]. The formulation and dose of estradiol will most commonly be oral estradiol 6 mg daily, but the specific medication route and dose will be at the discretion of the investigator, with all the goal in this arm to be suppression of ovulation and formation of the corpus luteum.

The first transvaginal ultrasound will be performed on menstrual cycle days 1–5 to provide a comparator to rule out later ovarian follicle development. At the first baseline visit, blood and urine sample will also be collected, as in the modified natural cycle.

Endometrial thickness and pattern will be assessed via ultrasound on days 11–16 of estradiol administration. As some literature suggests lower live birth rate with endometrial thickness < 7 mm, estradiol at the starting dose will be continued, and a repeat ultrasound will be scheduled in 2–7 days to assess whether endometrial thickness has reached 7 mm [30, 31].

If endometrium remains < 7 mm, estradiol (oral or patch or intramuscular) can be increased or vaginal estradiol may be added. The participant will begin progesterone administration once the endometrium reaches a thickness of ≥ 7 mm or after 28 days of estradiol administration even if the endometrium does not reach 7 mm. Although the estradiol doses administered are expected to suppress ovulation and formation of the corpus luteum, absence of the corpus luteum will be documented by ultrasound and serum progesterone level approximately 1.5 ng/ml or less, with the exact cut-off adjusted to local standards. The embryo transfer will be performed on the sixth day of progesterone administration, with flexibility of ± 2 days at the discretion of the investigator. Both estradiol and progesterone will be continued until the pregnancy test. If the participant is not pregnant, she will discontinue estradiol and progesterone at the time that failure to achieve pregnancy is determined. If she is pregnant, estradiol and progesterone will be continued typically without dose change until approximately 10 weeks gestation ± 2 weeks at discretion of the study physician.

### Outcome assessments and comparisons

The primary outcome will be the proportion of preeclampsia or maternal or fetal death in women with a viable pregnancy assigned to a modified natural cycle FET protocol compared to the proportion in women with a viable pregnancy assigned to a programmed FET protocol. Our secondary outcomes will be a comparison of the proportion of live births and the proportion of preeclampsia with severe features between the modified natural cycle FET and the programmed cycle FET. Finally, we will compare fetal growth in the two treatment arms measured at each trimester and other outcomes related to pregnancy success and infant health.

Data on the primary and secondary outcomes will be collected via obstetrical medical record abstraction from data collected on and after 20 weeks gestation through the end of pregnancy or live birth. Hypertensive disorders of pregnancy will be adjudicated by masked, trained obstetrician-gynecologists, who will use American College of Obstetricians and Gynecologists definitions for hypertensive disorders in pregnancy, which define preeclampsia as the development of hypertension (systolic blood pressure ≥ 140 mmHg and/or diastolic blood pressure ≥ 90 mmHg measured at least twice, 4 h apart) after 20 weeks gestation in a previously normotensive woman and proteinuria [32].

If a participant does not have proteinuria but has new onset hypertension, at least one of the following additional symptoms indicate the presence of preeclampsia: thrombocytopenia, new onset renal insufficiency, impaired liver function, pulmonary edema, and cerebral or visual symptoms. Additional criteria defining preeclampsia with severe features will be a systolic blood pressure of ≥ 160 mmHg and/or a diastolic blood pressure of ≥ 110 mmHg, or HELLP syndrome.

Outcome assessors will classify each case of hypertensive disorders according to the following designations. Early-onset preeclampsia occurs before 34 weeks gestation. Gestational hypertension will be defined as new-onset hypertension without proteinuria whereas chronic hypertension will be defined as hypertension diagnosed before 20 weeks or before conception. Chronic hypertension with superimposed preeclampsia will be defined as an increase of blood pressure in woman with chronic hypertension and new onset or worsening proteinuria or one of the features of severe preeclampsia. Eclampsia will be diagnosed if a woman with preeclampsia developed new-onset grand mal seizures.

It is important to note that new criteria could be utilized if the ACOG changes any of these definitions by the time of data analysis.

### Safety monitoring

Safety monitoring will be conducted actively through structured, close-ended questions posed to participants at each study visit and telephone contact from enrollment through 6 weeks post-partum. Participants will be asked about known and expected side effects related to the study treatment, any changes in overall health, new onset of symptoms, hospitalizations, any reactions to treatments, and any other adverse health effects.

Additional safety data will be gathered through medical record abstraction accumulated during follow-up. Obstetrical records will be requested for all participants released to obstetrical care. Information regarding any hospitalizations during the pre-natal period will be gathered from the telephone contacts during pregnancy.

For NatPro, adverse events are defined according to the Office of Human Research Protection guidance [33]. All adverse events will be recorded and reported annually in aggregate to the Johns Hopkins School of Medicine sIRB. The DSMB will receive a report of all adverse events by masked treatment group at each scheduled meeting. We use standard definitions for serious adverse events (SAEs) [33], except hospitalization for routine delivery is not considered an SAE in NatPro. Any pregnancy loss after 20 weeks gestation or neonatal death up to 6 weeks post-delivery will be accumulated as SAEs. All SAEs will be reported to the sIRB, NICHD, and the DSMB in an expedited manner.

### Sample size and power

We hypothesize that a lower proportion of women achieving a viable pregnancy with a modified natural cycle will experience preeclampsia, compared to women achieving a viable pregnancy with a programmed cycle. Because the primary outcome in NatPro is an obstetrical outcome, we calculated the sample size based on the assumption of an overall viable pregnancy rate of 50% over three FET cycles, as only those with a viable pregnancy are at risk for the primary outcome. A sample size of 358 pregnant women with a 1:1 allocation ratio to each treatment group will have 80% power to detect a difference of 8.3% for preeclampsia assuming 50% rate of viable pregnancy in both groups, a 12.8% proportion of preeclampsia in pregnant women using a programmed cycle FET, and 3.9% in women undergoing natural cycle FET (relative risk = 0.30), with a two-sided type I error proportion of 5%, using predicted proportion of preeclampsia based on data from vonVersen et al. [21]. Inflating by 10% to counter potential losses to follow-up results in a final sample size of 788 women or 394 women per treatment group. These calculations were performed in SAS version 9.3.

### Statistical methods

We will use intention-to-treat analyses for both the primary and secondary outcomes and will include all women who achieve a viable pregnancy following an FET for the primary outcome (preeclampsia) and all randomized women in the primary analysis for the secondary outcome (proportion of live births), in both cases analyzing according to the assigned treatment group. Participants, either women, fetuses, or both, who die will be counted as having the primary outcome in the primary analysis, to avoid death as a competing risk for experiencing the primary outcome.

The primary outcome analysis will be the comparison of proportions of preeclampsia or maternal or fetal death among women who achieve a viable pregnancy using Fisher’s exact test with a significance threshold of *p* = 0.049 (*p* value adjusted for interim analysis), adjusted for clinic and PGT status. We will also perform sensitivity analyses, including [1] a per-protocol analysis, [2] classifying participants with missing outcome data as having preeclampsia, [3] multiple imputation of preeclampsia assessment for missing outcomes, [4] a complete case analysis, [5] a restricted analysis of the first two treatment periods only, and [6] a stratified analysis to examine possible effects of clinical site and PGT status.

With the sample size described above, and assuming a projected cumulative live birth proportion on the order of 50–60%, we anticipate being able to describe live birth proportions to within approximately ± 5% (95% confidence interval). While we will likely not be able to conclude that the live birth proportion in the modified natural cycle is non-inferior to that in the programmed cycle, we will be able to establish the live birth proportions with sufficient precision to inform clinical decision making. The width of the 95% confidence interval does not change appreciably as the observed live birth proportion changes within the range of 40–70%.

Secondary analyses will also include an adjusted comparison of proportions of preeclampsia with severe features between treatment groups using Fisher’s exact test with a significance threshold of *p* = 0.05. Finally, we will compare fetal growth in the two treatment arms measured at each trimester using mixed effects logistic regression models with adjustment for covariates. Significance level will be set at the < 0.05 level with two-tailed tests. Statistical analyses will be performed using R statistical software and will be performed in duplicate by independent analysts.

## Discussion

### Pragmatic trial approach

NatPro was designed with a pragmatic approach to trial design to provide applicable evidence for informing clinical decision-making around FET protocol choice. While study procedures are standardized across study sites, NatPro interventions closely mirror current practices in programmed and natural cycle FET [27, 29, 34]. Site investigators are at liberty to apply discretion in the formulation of study medications, as in clinical practice. Additionally, sites will recruit women from a patient pool that would be seeking FET from their clinics under non-study scenarios. Sites are also a mix of academic and private practice locations in infertility insurance coverage mandated and non-mandated states, potentially diversifying the study population. Together, these design features will allow for real-world findings that can be streamlined with existing clinical practice.

### Design considerations and limitations

While NatPro has been carefully designed to address the question of whether or not a modified natural cycle will result in less risk for preeclampsia and fewer live births than a programmed cycle, there are some inherent limitations. The first limitation is the unmasking of the participants, clinic staff, site investigators, and principal investigators to treatment group assignments. Due to the differences in study procedures for each of the treatment arms, masking would be impractical and sham procedures could lead to undue burden on the study participants. To address this potential for bias, we have an independent, masked, outcome adjudication board to assess hypertensive disorders in pregnancy.

Second, we are analyzing women who achieve a viable pregnancy in each assigned treatment group for the occurrence of preeclampsia. We recognize that analyzing only those who have a viable pregnancy does not maintain the intended randomization of baseline characteristics. However, by including all randomized participants in the primary analysis, our results would be subject to potential bias if treatment was associated with the occurrence of pregnancy [35]. As such, we have planned a sensitivity analysis of the primary outcome comparing all randomized participants in each treatment group.

Finally, our study may face challenges in the retrieval of medical records for outcome ascertainment. Acceptance of medical release forms, timeliness of delivery, responsiveness of obstetrical offices, and receiving hospitals are a few of the anticipated problems [36]. Through trimester contacts, we hope to supplement medical record data and decrease loss to follow-up by gathering clinical data through structured questionnaires. In many cases, we anticipate that we will be able to access electronic medical records of the participant’s prenatal care and delivery. Together, these multiple forms of data collection will minimize missing data.

### Preeclampsia as primary outcome

The NatPro trial will be powered to detect a pregnancy outcome as opposed to only the outcome of clinical pregnancy or live birth. While we recognize the importance of pregnancy as an outcome in fertility trials, we were interested in investigating the potential impact of the choice of FET protocol and the health and well-being of women in the short and long terms. In consideration of the potential impact of maternal stress due to preeclampsia on metabolic disorders [37] and neuropsychiatric disorders [38], NatPro can leverage its rigorous statistical methods to produce informative study results, which can help in the development of a larger observational cohort study of long-term maternal effects to preeclampsia.

## Trial status

NatPro was registered on clinicaltrials.gov September 16, 2020 (NCT04551807). This design and methods manuscript is based on NatPro Protocol Version 1.2, December 16, 2020. All future protocol amendments are subject to review and approval by the Johns Hopkins School of Medicine sIRB (IRB00214688). Recruitment began in August 2020 and is expected to complete by July 1, 2023.

## Supplementary Information

**Additional file 1: Supplemental Table 1.** Eligibility Criteria

## Data Availability

The NatPro study team and the funder, the National Institutes of Child Health and Human Development (NICHD), will have access to the cleaned dataset at the end of the study. Where participants provide consent, anonymized data will be made available through the NICHD Data and Specimen Hub (DASH).

## Abbreviations

ART: Assisted reproductive technology
FET: Frozen embryo transfer
HELLP: Hemodialysis, elevated liver enzymes, and low platelets syndrome
DSMB: Data and safety monitoring board
DCC: Data coordinating center
LH: Luteinizing hormone
SAE: Serious adverse event
