# Supplementary material for: Natural vs. programmed cycles for frozen embryo transfer: study protocol for an investigator-initiated, randomized, controlled, multicenter clinical trial
Source: Trials. 2021 Sep 27;22:660. doi: 10.1186/s13063-021-05637-3 (PMC8477459; doi:10.1186/s13063-021-05637-3)
Supplement: Supplementary file 1 — Additional file 1: Supplemental Table 1. Eligibility Criteria [file 13063_2021_5637_MOESM1_ESM.docx]

## Supplemental Table 1: Eligibility Criteria

| **Inclusion criteria**  To be eligible, subjects must meet all these criteria:   - Age 18-39 years at the time that embryos were created if no PGT testing was performed. If PGT testing was performed and indicates that an embryo is euploid, the patient can be included if she was age 18-41 years at the time that the embryo was created. - Age 18-41 years at the time of randomization as advancing age itself becomes a significant risk for preeclampsia - Normal uterine cavity assessed by saline infusion sonohysterogram, hysterosalpingogram, or hysteroscopy within 1 year of enrollment, and repeated at the discretion of the investigator - Regular menstrual cycle length (approximately 24-35 days) indicative of ovulatory cycles. - Willing to undergo elective single embryo transfer - Body Mass Index<=40 - If Body Mass Index is over 30 or individual has other risk factors for diabetes, normal hemoglobin A1C - Prior to enrollment, participant will have at least one vitrified blastocyst with euploid result by pre-implantation genetic testing (PGT-A) or at least one vitrified blastocyst of fair or better morphologic quality if no PGT-A results are available. - Willingness to be randomized to either a modified natural or programmed cycle, with a willingness to administer daily intramuscular progesterone in oil if assigned to the programmed cycle. - Normal thyroid stimulating hormone (TSH) , according to local laboratory standards, within one year of study enrollment and repeated at the discretion of the investigator. Use of thyroid medication is permitted. |
| --- |
| **Exclusion criteria**  To be eligible, subjects must not meet any one of these criteria:   - - Medical contraindication to pregnancy   - Embryos created using donor oocytes   - Embryo donation   - Gestational carrier - Reciprocal IVF (one female partner carrying pregnancy, other female partner as source of eggs)   - Embryos created from frozen oocytes   - Recurrent implantation failure defined as no clinical pregnancy with ≥2 prior consecutive embryo transfers unless patient also had successful live birth with IVF   - Anti-phospholipid antibody syndrome or rheumatologic disease requiring chronic systemic medications   - Uncontrolled diabetes mellitus   - History of >1 pregnancy loss in the second or third trimester   - Uncontrolled hypertension   - Untreated hydrosalpinx (i.e., hydrosalpinx in situ which has not been ligated or removed)   - Physician recommendation to perform the embryo transfer outside of the timing specified by the protocol   - Contraindication to any medication which must be used in preparation for the frozen embryo transfer (i.e., estradiol, progesterone, hCG). |
